# Chagasic megacolon: enteric neurons and related structures

**DOI:** 10.1007/s00418-014-1250-x

**Published:** 2014-07-25

**Authors:** Samir Jabari, Enio C. de Oliveira, Axel Brehmer, Alexandre B. M. da Silveira

**Affiliations:** 1 Institute of Anatomy I, University of Erlangen-Nuremberg, Krankenhausstr. 9, 91054 Erlangen, Germany; 2 Department of Surgery, Medical School, Universidade Federal de Goiás, Goiânia, 74.605-020 Brazil; 3 Human Anatomy Sector, ICBIM, Universidade Federal de Uberlândia, Uberlândia, MG 38.400-902 Brazil

**Keywords:** Chagas, Enteric nervous system, Hirschsprung, Megacolon, Vasoactive intestinal peptide

## Abstract

Megacolon, the irreversible dilation of a colonic segment, is a structural sign associated with various gastrointestinal disorders. In its hereditary, secondary form (e.g. in Hirschsprung’s disease), dilation occurs in an originally healthy colonic segment due to an anally located, aganglionic zone. In contrast, in chronic Chagas’ disease, the dilated segment itself displays pathohistological changes, and the earliest and most prominent being found was massive loss of myenteric neurons. This neuron loss was partial and selective, i.e. some neurons containing neuronal nitric oxide synthase and/or vasoactive intestinal peptide (VIP) were spared from neuron death. This disproportionate survival of inhibitory neurons, however, did not completely correlate with the calibre change along the surgically removed, megacolonic segments. A better correlation was observed as to potentially contractile muscle tissue elements and the interstitial cells of Cajal. Therefore, the decreased densities of α-smooth muscle actin- and c-kit-immunoreactive profiles were estimated along resected megacolonic segments. Their lowest values were observed in the megacolonic zones itself, whereas less pronounced decreases were found in the non-dilated, transitional zones (oral and anal to dilation). In contrast to the myenteric plexus, the submucosal plexus displayed only a moderate neuron loss. Neurons co-immunoreactive for VIP and calretinin survived disproportionately. As a consequence, these neurons may have contributed to maintain the epithelial barrier and allowed the chagasic patients to survive for decades, despite their severe disturbance of colonic motility. Due to its neuroprotective and neuroeffectory functions, VIP may play a key role in the development and duration of chagasic megacolon.

## Structural components of the human enteric nervous system

**Fig. 1:**
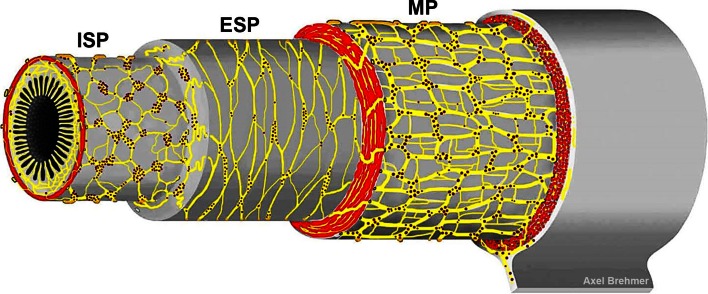
Schematic drawing of the three ganglionated plexus of the human enteric nervous system, depicted in layer preparation (*red*: cross-sectioned muscle layer profiles). The different network architectures were approached from numerous original small and large intestinal wholemount specimens. Between the three ganglionated plexus, there are abundant interconnecting strands. Neurons outside these main ganglionated plexus are common. *MP* myenteric plexus, *ESP* external submucosal plexus, *ISP* internal submucosal plexus

The overwhelming majority of enteric neuronal cell bodies lie, together with enteric glial cells, in ganglionated nerve networks, the myenteric and the submucosal plexus. These differ in location and network architecture (Fig. 1) as well as in neuronal composition (see below).

Situated between the longitudinal and the circular muscle layer is the myenteric plexus (Auerbach 1862). Its main, primary network is commonly monolayered and extends from the upper oesophageal to the internal anal sphincter.

Most human myenteric neurons are either nitrergic or cholinergic (Murphy et al. 2007; Beck et al. 2009). Like in other species (best known from the guinea pig, Furness 2006), human nitrergic myenteric neurons are suggested to be either descending inter- and/or inhibitory motor neurons (Grider 1993). They are uniaxonal with spiny shaped dendrites (spiny type I morphology; Brehmer et al. 2004a; Lindig et al. 2009). Besides their reactivity for the nitrergic marker neuronal nitric oxide synthase (nNOS), some of them co-stain immunohistochemically for vasoactive intestinal peptide (VIP; Brehmer et al. 2006; Schuy et al. 2011). In contrast, cholinergic myenteric neurons, immunoreactive for the common choline acetyltransferase (cChAT), include primary afferent, inter-, and excitatory motor neurons as mainly known from the guinea pig (Furness 2006). Although we have characterized morpho-chemically some non-nitrergic myenteric neuron types in human intestines (Brehmer et al. 2004a, b, 2005), our knowledge of diverse enteric neuron types in the human gut is still fragmentary (Brehmer 2006).

The submucosa of larger mammals harbours a two- or even multilayered plexus (Meissner 1857) which is restricted to the small and large intestines. In human, two ganglionated submucosal plexus can be distinguished (Brehmer et al. 2010), primarily based on their locations within the submucosal layer and their network architectures. The internal submucosal plexus (reassigned to Meissner) lies in the inner half of the submucosa and is frequently multilayered. The external submucosal plexus (Schabadasch 1930) is situated near the inner border of the circular muscle layer and is rather mono-(occasionally two-) layered. As to their neuronal compositions, we have hitherto found only quantitative differences between the human internal and external submucosal plexus (see below). This is in contrast, e.g., to the pig, where clear qualitative differences between the two submucosal plexus were described (Stach 1977; Timmermans et al. 2001; Kapp et al. 2006).

The great majority of human submucosal neurons is cholinergic. Submucosal nitrergic neurons represent a small minority and are partly even absent from the submucosal plexus (Timmermans et al. 1994; Beyer et al. 2013; Beuscher et al. 2014). Among the cholinergic neurons, there are two separate populations that commonly amount for up to 80 % of human submucosal neurons. The larger population is, in the colon, co-immunoreactive for VIP and calretinin (CALR), and the smaller one for somatostatin (SOM) and, partly, substance P (SP; Kustermann et al. 2011; Beyer et al. 2013; Beuscher et al. 2014). The two populations differ also morphologically: VIP/CALR-neurons are dendritic, whereas SOM/SP-neurons are non-dendritic and (pseudo)uniaxonal. Both submucosal neuron populations may have multiple functions (see below). Among these, VIP/CALR-neurons may act as secretomotor neurons (Karaki and Kuwahara 2004; Farthing 2006; Margolis and Gershon 2009; Chandrasekharan et al. 2013; Beuscher et al. 2014), and SOM/SP-neurons may be primary afferent (Farthing 2006; Kustermann et al. 2011; Beyer et al. 2013).

## Megacolon: secondary versus primary

**Fig. 2:**
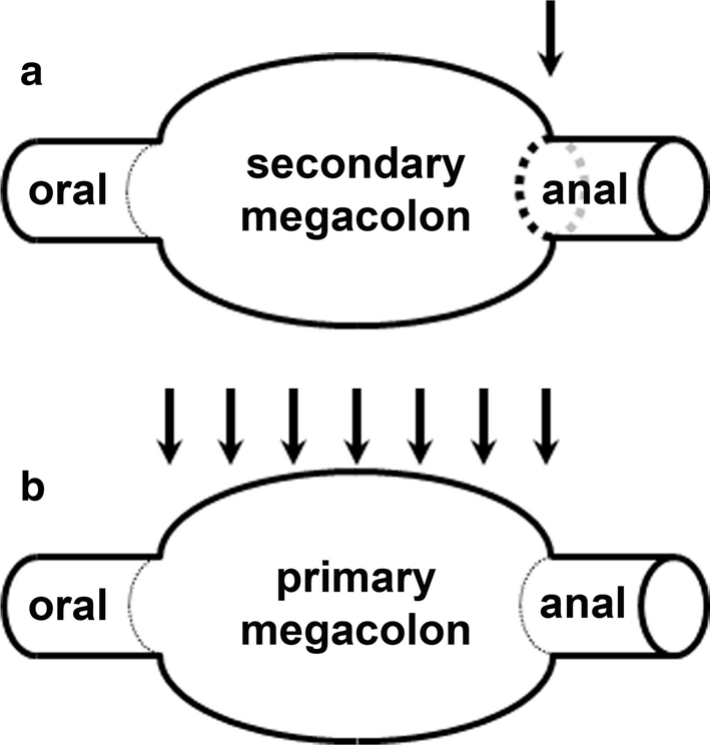
Two alternate ways for the development of megacolon. **a** Secondary megacolon (e.g. in Hirschsprung’s disease) is due to permanent stenosis of an aganglionic segment (*arrow*) followed by stasis and secondary inflation of an originally healthy segment. **b** Primary megacolon (e.g. in Chagas’ disease) is due to inflation of the originally diseased segment itself (*arrows* indicate the zone of an acquired hypoganglionosis)

Megacolon is the chronic dilation of a colonic segment, irrespective of the location of its origin (Fig. 2). Already, after its first description by Hirschsprung (1888; cited after Howard 1972), several early authors suspected the cause of this congenital form not in the ‘megacolon’ itself but in the absence of enteric neurons (aganglionosis) in the anally located zone (Bodian et al. 1949; Truelove 1966; Howard 1972). As a consequence, the smooth musculature of the affected segment is in a permanent state of contraction. The resulting stenosis causes stasis of ingesta. Subsequently, adaptive transformation of the orally located, originally healthy colonic segment toward a *secondary megacolon* (Bodian et al. 1949) evolves.

In contrast, enteric neurodegeneration of an originally healthy (normoganglionic) segment becomes manifest in at least 20 % of patients suffering from Chagas’ disease (Köberle 1968). During its chronic phase, (my)enteric neuron loss and irreversible dilation occur in one and the same (frequently colonic) gut segment. Since cause and effect are at the same place, this dilation may be termed as *primary megacolon*. At the latest since Köberle, (my)enteric neuron loss was commonly considered as the cause for chagasic megacolon: ‘…denervation is the *absolutely indispensable* element for the appearance of these (chagasic megacolonic) syndromes…’ (Köberle 1968).

During the 1960s, when Köberle and others searched for pathohistological alterations in colonic (and other) megasyndromes, the enteric nervous system (ENS) awaited its conceptual renaissance (Brehmer et al. 1999). Primarily based on works in the guinea pig, the unique position of the ENS within the peripheral nervous system as well as the complexity of its internal organization and external relationships with surrounding tissues and systems was realized (reviewed in Furness and Costa 1987; Furness 2006). These advances in our understanding of the ENS were scarcely accompanied by advances in the explanation of the development of (chagasic) megacolon.

The above, classical explanations for Hirschsprung’s megacolon on the one hand (regional absence of enteric neurons leads to permanent *contraction* of adjacent smooth muscle) and Chagas’ megacolon on the other hand (regional absence of enteric neurons leads to permanent *dilation* of adjacent smooth muscle) are contradictory. In addition, a further, less considered phenomenon requires explanation. The ENS regulates not only motor but also mucosal functions, a vital one being the maintenance of the epithelial barrier of the mucosa. In patients suffering from chagasic megacolon for decades, this barrier should be largely intact which indicates that certain components of the ENS should be undamaged as well. This led us to analyse the fate of different subgroups of enteric neurons and of some of their related structures in chagasic megacolonic segments.

## Evaluation of enteric nervous tissue, interstitial cells of Cajal, and smooth musculature

The enteric nervous tissue consists of neuronal and glial components. To estimate quantitatively their representation in our wholemount and section specimens, we used general and specific immunohistochemical markers.

As marker for demonstrating neuronal cell bodies, the human neuronal protein Hu C/D (HU; Phillips et al. 2004; Ganns et al. 2006) is well established. In contrast, an immunohistochemical pan-axonal marker is, to our knowledge, not generally established. Here, we used synaptophysin (SYN) which is specific for synaptic vesicles (Wiedenmann and Franke 1985; Dzienis-Koronkiewicz et al. 2005). Furthermore, specific neuronal markers were applied (see above: CALR, cChAT, nNOS, SOM, VIP).

As marker for the evaluation of changes of the glial component of the enteric nervous tissue, we used the calcium-binding protein S100 (Ferri et al. 1982; da Silveira et al. 2009). In addition to their ‘classical’ role as pure supporting cells, a number of specialized functions were recently attributed to enteric glial cells, e.g. support of the epithelial barrier function, modulation of neurotransmission, neurogenesis, etc. (Gulbransen and Sharkey 2012).

Interstitial cells of Cajal (ICCs) have been demonstrated by immunolabelling of the c-kit receptor (Huizinga et al. 1995; Vanderwinden et al. 1996; Takaki 2003; Blair et al. 2012), which is located in both ICCs and mast cells but not in enteric neurons (Maeda et al. 1992). ICCs are considered as pacemakers of motility and mediate the input of neurons to the muscularis propria (Thuneberg 1982; Rumessen and Thuneberg 1996; Ward et al. 2004) although this role has also been disputed (see below).

To enable conclusions as to the amount of potentially contractile smooth muscle tissue within the thickened muscle layers, we applied α-smooth muscle actin (SMA) as marker for gut musculature (Wedel et al. 2006). In detail, we estimated the smooth muscle density as stained SMA-profile area related to the muscle layer area in cryosections through the gut wall.

## Chagasic megacolon: myenteric neurons and intramuscular nerve fibres

Pathohistological changes obvious in myenteric wholemount specimens of chagasic/megacolonic tissues appear as prototypic signs of degeneration which were most distinct in the dilated megacolonic and the anal (non-dilated) region (Jabari et al. 2011). Similar alterations have earlier been demonstrated, by applying various methods, in colonic specimens derived from quite different diseases and from aged colon (De Biscop 1949; Smith 1972; Schuffler et al. 1978, 1985; Schuffler and Jonak 1982; Krishnamurthy et al. 1985; Phillips et al. 2003; Hanani et al. 2004). Instead of myenteric ganglia containing numerous neurons, networks of bulky fibre bundles, comb-like plexus structures, and ‘empty’ ganglia without nerve cells were observed. These general signs of neurodegeneration were paralleled by a decrease of S100-immunoreactive glial elements which may leave the nerve elements unprotected against inflammatory damage (da Silveira et al. 2009; Jabari et al. 2013).

Neuron loss in Chagas’ disease is thought to begin in the acute phase after infection with the protozoon *Trypanosoma cruzi* by direct action of the pathogen, whereas it continues in the chronic phase by (auto)immunologic reactions of the organism (Köberle 1968; Hudson and Hindmarsh 1985; Dutra et al. 2009). However, the parasite does not disappear completely from the afflicted host in most cases (Clayton 2010). During the decades of the chronic phase of Chagas’ disease, a megacolon becomes clinically manifest by slowly progressive constipation (de Oliveira et al. 1998). To transform a previously occurring ‘functional’ motility dysbalance into a permanent megacolon, Köberle (1968) and Meneghelli (2004) suggested that loss of more than half of the colonic enteric neurons is required. Meneghelli (2004) found a 50 % loss of myenteric neurons in small intestinal samples displaying no mega-syndrome. Whereas megaesophagus occurs almost as frequently as megacolon in chronic Chagas’ disease, ‘megas’ of other intestinal regions are rare (Köberle 1968). [By far the most frequently affected organ in chronic Chagas’ disease is the heart (Köberle 1968)].

**Fig. 3:**
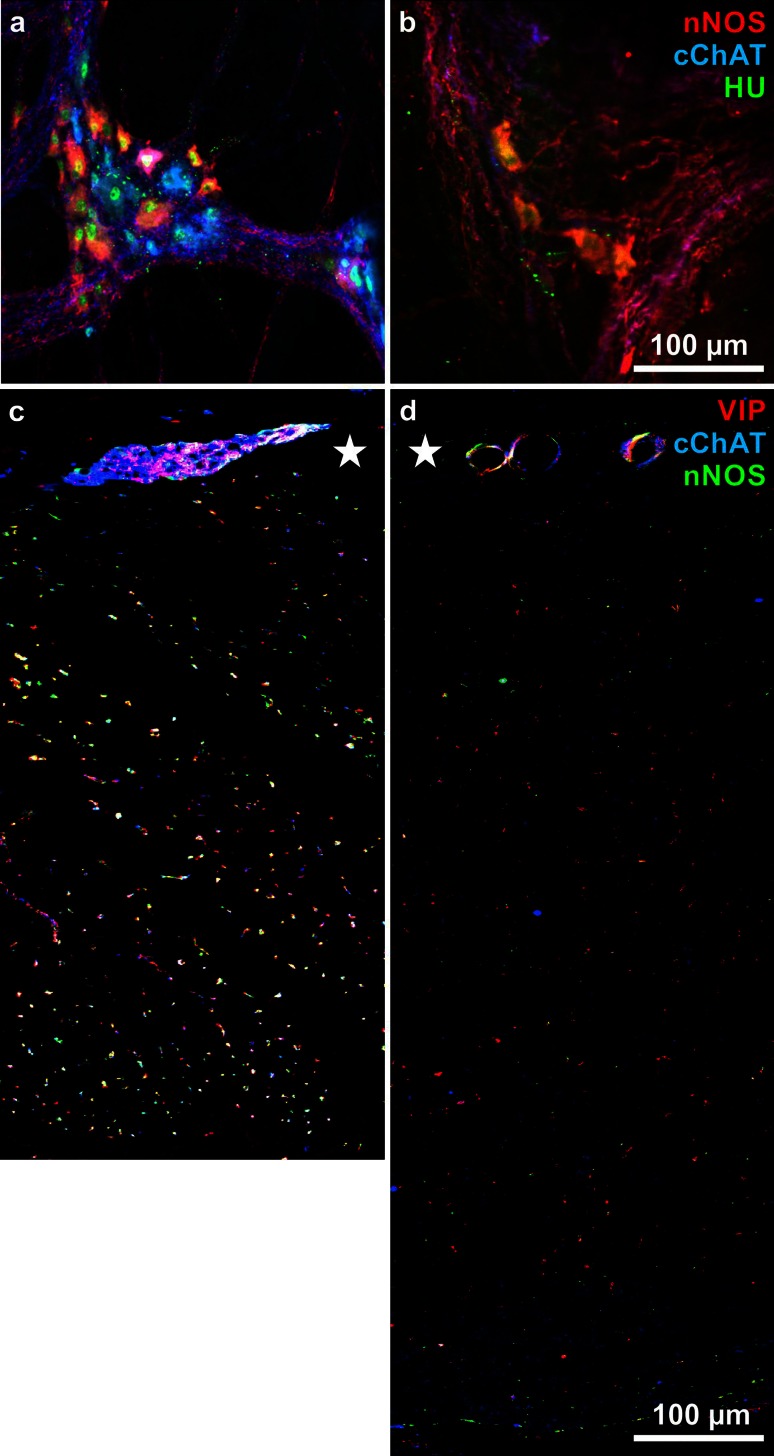
**a, b** Myenteric ganglia in wholemounts immunohistochemically triple stained for nitric oxide synthase (nNOS, *red*) common choline acetyl transferase (cChAT, *blue*) and the human neuronal protein Hu C/D (HU, *green*). **a** is from a non-chagasic/non-megacolonic (‘control’), note the numerical balance between *red* and *blue* neurons, both with *green* nuclei. **b** is from a chagasic/megacolonic specimen, note the loosened structured ganglionic architecture with few, *red* neurons. **c, d** Circular muscle layers with myenteric ganglia (*asterisks*) in cross sections stained for vasoactive intestinal peptide (VIP, *red*), cChAT (*blue*), and nNOS (*green*). **c** control specimen, **d** chagasic/megacolonic specimen. Note the increased layer thickness and the decreased nerve fibre density in (**d**) in contrast to (**c**)

Extensive myenteric neuron loss in chagasic megacolon was shown in a number of studies (Köberle 1968; Fernandez et al. 1992; Adad et al. 2001; Meneghelli 2004; Iantorno et al. 2007). Own results generally confirmed these findings and additionally demonstrated parallel decrease of nerve fibre density in the circular and longitudinal muscle layers (Jabari et al. 2011, 2012b). This was demonstrated both by applying general neuronal and glial (SYN, S 100) as well as specific neuronal markers (see below). Besides general signs of neurodegeneration (see above), we additionally found the few, remaining neurons to be frequently deformed and hyper- or atrophic and to be mostly immunoreactive for nNOS and/or VIP (Fig. 3). Co-application of the pan-neuronal marker HU enabled us to discriminate the relative increase of the nitrergic/VIP-ergic subpopulation from its absolute decrease (Jabari et al. 2011 in contrast to Ribeiro et al. 1998). The fraction of cholinergic neurons and nerve fibres, however, was disproportionately decreased. Different studies in non-chagasic/non-megacolonic samples revealed between 51 and 56 % nitrergic myenteric neurons (Beck et al. 2009; Jabari et al. 2011). In contrast, in chagasic/megacolonic tissues, this proportion increased up to 86 % (Jabari et al. 2011). Even more conspicuously, the fraction of nitrergic neurons co-localizing VIP increased disproportionately. In non-chagasic/non-megacolonic colon, nNOS/VIP-neurons amounted to only 9 % (Schuy et al. 2011), whereas in chagasic/megacolonic samples, up to 51 % (Jabari et al. 2012b). Generally, these changes in ganglionic composition were paralleled by changes in intramuscular nerve fibre spectrum. Besides a general reduction in nerve fibre density, nerve fibres immunoreactive for nNOS or VIP or both were increased. For example, in control colonic circular muscle, 68 % of nerve fibres were nNOS- and/or VIP-reactive, and this proportion increased from 75 % (in the oral transitional zone) via 84 % (megacolonic zone) up to 87 % (anal zone; Jabari et al. 2012b). In mice infected with *Trypanosoma cruzi*, nitrergic nerve elements were similarly spared from extensive loss (Ny et al. 1999). Also, with ageing, a relative, although less pronounced increase of nitrergic neurons was described in both animals and humans (Santer 1994; Phillips et al. 2003; Bernard et al. 2009).

Reasons for this partial, selective survival of nitrergic and VIP-ergic nerve elements were discussed (Jabari et al. 2011, 2012b) and will be dealt with below. Consequences of the prevalence of neurons and nerve fibres containing these two inhibitory neurotransmitters (Grider 1989, 1993) seem to allow a casual explanation of the development of a permanently dilated ‘megacolon’. However, analysing also the transitional zones directly oral and anal to the dilated, megacolonic segment, only the findings of the oral zone (displaying a less pronounced destruction and loss of cholinergic nerve elements) were in line with this explanation.

In contrast, in the anal zone, the dysbalance between inhibitory (VIP, nNOS containing) and excitatory (cholinergic) nerve elements was most pronounced toward the former; however, this region was not dilated. In other words, the extent of changes demonstrated immunohistochemically by neuronal and glial markers corresponded with the change of calibre along the surgically removed chagasic megacolon only in the oral transitional and the megacolonic zones. They did not correspond in the anal transitional zone. Here, the change of neuro-immunohistochemical parameters toward inhibitory elements is not accompanied by dilation. This suggests that the neuronal dysbalance alone is not sufficient for explaining the chronic dilation, and additional factors must be taken into account.

## Chagasic megacolon: smooth muscle and interstitial cells of Cajal (ICC)

**Fig. 4:**
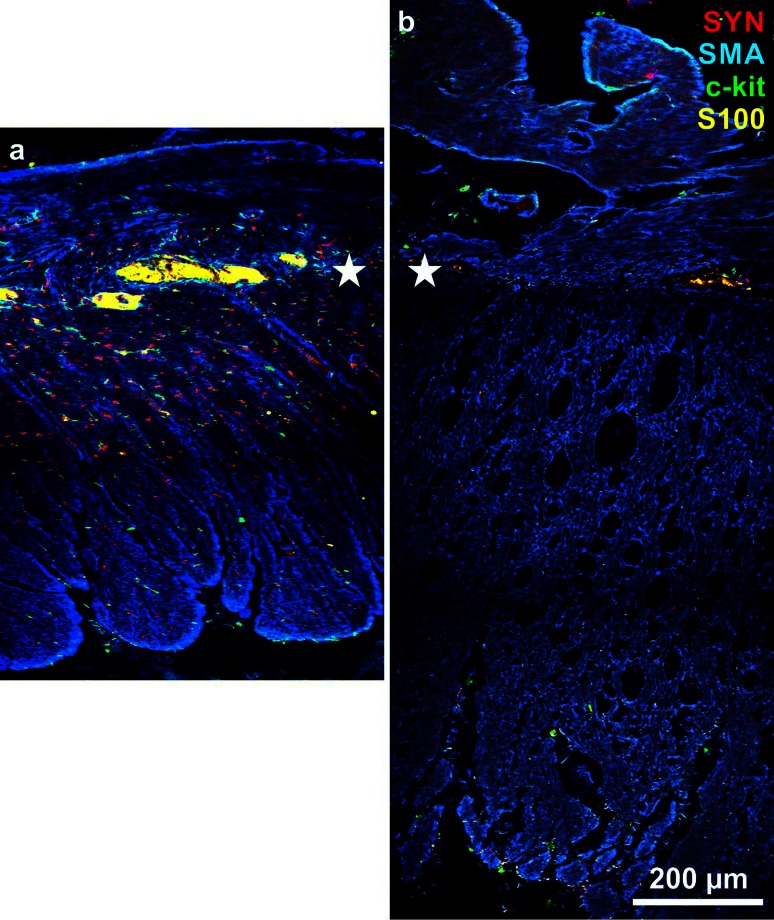
Cross sections through the muscle coats (adjusted to the plane of the myenteric plexus, *asterisks*) immunohistochemically quadruple stained for synaptophysin (SYN, *red*), α-smooth muscle actin (SMA, *blue*), c-kit (*green*), and S100 (*yellow*). **a** control specimen, **b** chagasic/megacolonic specimen. Note in (**b**) in contrast to (**a**): the increased thickness of both muscle layers (*blue*), the loosened architecture of the muscle tissue (*blue*), and the decreased density of the other three markers (for details see Jabari et al. 2013)

Both the external and the mucosal muscle layers are remarkably thickened in chagasic megacolon (Köberle 1968). Permanent dilation of a (colonic) gut segment is accompanied by thickening of its wall. Initially, thickening predominates over dilation, whereas later, as a sign of decompensation, the gut wall distends with the muscle layers becoming thinner (Köberle 1968). Adad et al. (2012) described muscle area thickening in sections of the muscularis propria in chagasic megacolon increasing as much as five times compared to controls. These authors concluded that the development of the dilation is accompanied by muscular hypertrophy. But muscle layer thickening is not only due to an increase of muscle tissue but also to proliferation of connective tissue. Massive fibrosis has been found in chagasic megacolon (Pinheiro et al. 2003; da Silveira et al. 2007; Iantorno et al. 2007; Jabari et al. 2014). Consequently, in the thickened muscle layers of the dilated megacolonic zones, we found a decreased density of SMA together with a loosened architecture of the SMA-stained circular and longitudinal layers (Fig. 4; Jabari et al. 2013). Decreased density of SMA has been found also in other motility disorders, together with a strikingly changed architecture of musculature (Knowles et al. 2004; Wedel et al. 2006). The decreased density of SMA and thereby the reduction of contractile elements in the megacolonic zone may be a sign of the massive impairment of motility. Besides, there are also reports that no or non-significant deficiencies of smooth musculature morphology occur in intestinal pseudo-obstruction, Hirschsprung’s disease, and idiopathic intractable constipation (Gamba et al. 2004; Knowles et al. 2004; van den Berg et al. 2009).

A decrease of ICCs has been described in chagasic megacolon (Hagger et al. 2000; Matsuda et al. 2009; Adad et al. 2012). Although these cells are seen as contributors in the development of numerous motility disorders (Streutker et al. 2007; Rolle et al. 2007; Adachi et al. 2008; Sanders et al. 2010), their function especially in human gastrointestinal motility is unproven (Sanders et al. 2012).

The contraction of gastrointestinal smooth muscle is supposed to be generated by the common action of at least three electrically coupled cell types, i.e. ICCs, ‘non-ICC-interstitial cells’ (being immunoreactive for the platelet-derived growth factor receptor α), and smooth muscle cells. Enteric neurons, hormones, and paracrine substances modulate their contraction (Sanders et al. 2012). Obviously, in chagasic and Hirschsprung’s megacolon, the balance between these components is disturbed in different ways (see above and Fig. 2). However, in Hirschsprung’s (Gfroerer and Rolle 2013) and Chagas’ megacolon (see above), most studies found reduced numbers of ICCs in the hypo- or aganglionic segments. One exception is the study of Horisawa et al. (1998) who found no difference in ICC distribution between aganglionic (Hirschsprung) and intact descending colon. In chagasic megacolon, a close topographical correlation between the change of calibre along the resected specimens and the changes of parameters was observed related to smooth muscle and ICC densities (Jabari et al. 2011, 2012a, b, 2013). Values were lower in dilated and megacolonic, and were higher in non-dilated oral and anal zones (Jabari et al. 2013). That is, decrease in muscle tissue and ICC densities is topographically directly linked with the extent of the mega-syndrome. Thus, changes of these two parameters provide a more consistent explanation for the change of the colonic circumference.

## Chagasic megacolon: submucosal neurons and mucosal nerve fibres

Most studies dealing with chagasic megacolon focused on structures related to motility, and only few presented counts of submucosal neurons in chagasic gut segments (Costa and De Lima Filho 1964; Meneghelli 2004; Iantorno et al. 2007). Our own counts in chagasic/megacolonic submucosal plexus principally confirmed the results of the earliest authors (Costa and De Lima Filho 1964), i.e. in contrast to the massive myenteric neuron loss, the submucosal neuron loss was rather moderate (Jabari et al. 2012a). However, there is a selective loss of neurons also in the two submucosal plexus. Here, neurons co-immunoreactive for CALR and VIP were spared (Jabari et al. 2012a). In the healthy, colonic external and internal submucosal plexus, at the most one-third of neurons is VIP-negative and these neurons degenerated almost completely in chagasic megacolon. Among these VIP-negative neurons, the largest and best known population is the SOM (ChAT/SP)-immunoreactive one (Beyer et al. 2013). Consequently, mucosal nerve fibres immunoreactive for SOM were hardly found, whereas VIP(/CALR)-positive nerve fibres were, though reduced in density, present throughout all megacolonic and transitional zones (Jabari et al. 2012a). Within the mucosa, the muscularis mucosae and the lamina propria displayed diverging changes as to the megacolonic and the anal segments. The lamina propria showed highest layer thickness and lowest densities of nerve fibres, glia, and smooth muscle tissue in the dilated portion itself. In contrast, in the muscularis mucosae, the layer thickness was highest and the nerve/glia densities were lowest in the anal segment thus paralleling results of the (external) muscle coat (Jabari et al. 2014).

Considering the chronic, decades lasting phase of Chagas’ disease, it is evident that some vital mucosal structures must be maintained. One of these is the epithelium whose barrier function requires, e.g. cell proliferation and expression of tight junction proteins. Both depend on the action of VIP-ergic submucosal neurons (Neunlist et al. 2003; Toumi et al. 2003). If these VIP-neurons would have died in the course of Chagas’ disease, we suggest that these patients could not have survived for decades. In contrast, considering the almost total loss of SOM-neurons in chagasic megacolon, this population seems not to be of vital importance.

## Vasoactive intestinal peptide (VIP)

**Fig. 5:**
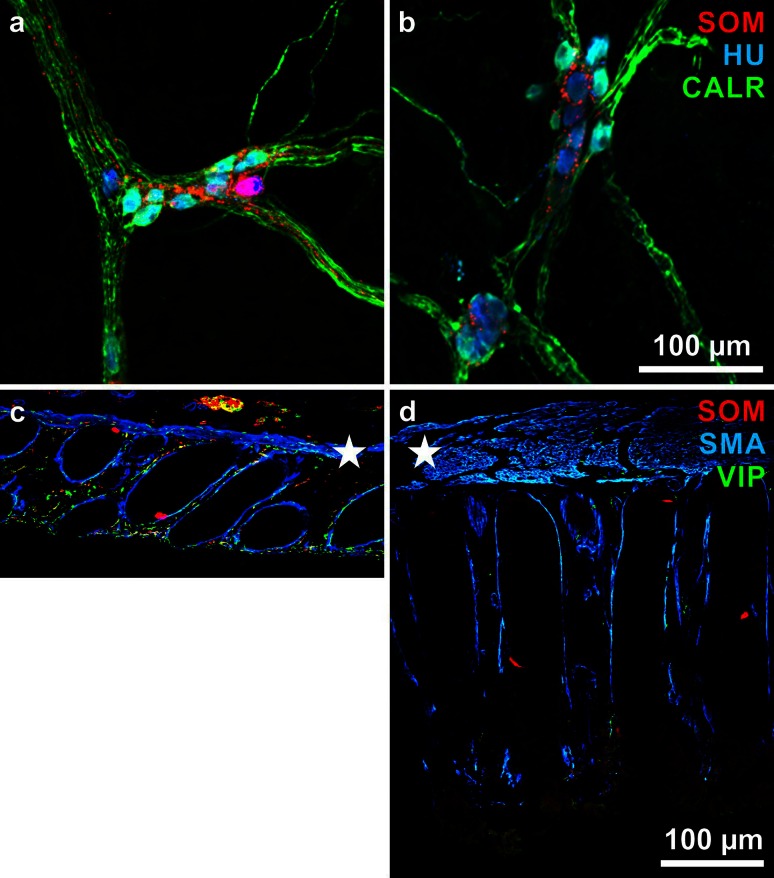
**a, b** External submucosal ganglia in wholemounts immunohistochemically triple stained for somatostatin (SOM, *red*), the human neuronal protein Hu C/D (HU, *blue*), and calretinin (CALR, *green*). **a** is from a non-chagasic/non-megacolonic (‘control’), **b** from a chagasic/megacolonic specimen. Note that in both cases, the ganglia display quite similar appearance; in (**b**), there is no *red* (SOM-positive) neuron. **c, d** Mucosal layers in cross sections (with adjusted muscularis mucosae, *asterisks*) triple stained for SOM (*red*), α-smooth muscle actin (SMA, *blue*), and vasoactive intestinal peptide (VIP, *green*). Note the increased thickness and the decreased nerve fibre density with a complete loss of red (SOM-reactive) nerve fibres in (**d**) in contrast to (**c**). SOM-positive enteroendocrine cells (*red* patches) are present in both (**c**) and (**d**)

The difference in proportions of VIP-neurons in the respective plexus reflects the difference in the numbers of surviving neurons in chronic Chagas’ disease. That is, VIP(/nNOS)-neurons are a minority in the healthy myenteric plexus—few myenteric neurons survive in chagasic megacolon (Fig. 3). In contrast, VIP(/CALR)-neurons are a majority in the healthy submucosal plexus—a considerable number of submucosal neurons survive (Fig. 5). Above, we discussed our suggestions as to quite different consequences of the survival of myenteric and submucosal VIP-neurons. The myenteric VIP-neurons may contribute to the development of the megacolon, whereas the submucosal VIP-neurons may enable the patients to survive by maintaining the epithelial barrier.

One reason for survival of these neurons may be VIP itself. It is well established that this peptide is neuroprotective (Ekblad and Bauer 2004; Pozo and Delgado 2004; Brenneman 2007; Arranz et al. 2008; Ben-Horin and Chowers 2008). Additionally, also nitric oxide was able to promote enteric neuronal survival in culture (Sandgren et al. 2003). We suggest that VIP may protect both the submucosal VIP-neurons, acting on epithelial cells, and the myenteric VIP-neurons, acting together with nNOS as inhibitory motor neurons.

In conclusion, we suggest that during megacolon formation in chronic Chagas’ disease, neuron loss alone does not explain completely the loss of motility and the subsequent calibre change. Other cells and tissues (e.g. ICCs, smooth muscle cells) may be primarily damaged as well. As to the survival of patients despite the obvious changes in the enteric nervous system, VIP may play a key role due to its neuroprotective and neuroeffectory functions.
